# Characteristics of the Construction Industry in Developing Countries and Its Implications for Health and Safety: An Exploratory Study in Ghana

**DOI:** 10.3390/ijerph17114110

**Published:** 2020-06-09

**Authors:** Elijah Frimpong Boadu, Cynthia Changxin Wang, Riza Yosia Sunindijo

**Affiliations:** Faculty of Built Environment, The University of New South Wales, Sydney, NSW 2052, Australia; b.frimpong@unsw.edu.au (E.F.B.); cynthia.wang@unsw.edu.au (C.C.W.)

**Keywords:** construction industry, health and safety, developing countries, characteristics

## Abstract

From both practical and theoretical perspectives, understanding the health and safety (H&S) implications of the characteristics and foundation upon which the construction industry in developing countries is built and operates is essential for H&S management within the industry. While many studies have provided evidence of factors affecting construction H&S in developing countries, none has fully considered the H&S implications of the industry’s characteristics. The current study thus examined how the peculiar characteristics of the construction industry in developing countries impact on the industry’s H&S management. Data were collected using questionnaire surveys from construction industry professionals in Ghana. Nine distinct characteristics were identified and ranked, as well as their relationships and statistical significance determined through correlation and analysis of variance (ANOVA), respectively. The findings showed that these characteristics of the construction industry in developing countries, particularly the lack of skilled and educated workforce, reliance on labour intensive methods and lack of single regulatory authority, present huge challenges to the management of H&S. Accordingly, this research recommended strategic interventions which are tailored towards the context of the industry’s characteristics. With the construction industry in developing countries exhibiting similar characteristics, the findings of this research can serve as a framework for country-specific study. The study contributes to the broader H&S performance improvement research in developing countries by throwing light on the characteristics of the industry that pose challenges to H&S performance.

## 1. Introduction

The construction industry significantly contributes to economic growth and social development of nations. In Ghana, the construction industry is the second largest contributor to Gross Domestic Product (GDP) with 13.7%, just behind crops [1]. Other studies have established that significant relationships exist in one way or another between the rate of growth of the construction industry and the rate of macroeconomic growth of developing countries [2]. Other industrial sectors of the economy depend on the products of the construction industry to produce other goods and services. For example, the construction of roads, infrastructure, water supply and electricity lines can boost the production of goods and services while creating employment for the people. In a developing country like Ghana where unemployment is high, the construction industry is an important source of jobs for the unemployed and/or unskilled [3]. The Ghana Statistical Service in its 2015 published labour force report revealed that the construction industry employs over 600,000 workers, amounting to about 7% of the working population in Ghana [4]. The number may actually be higher in reality because the number of informal workers is not recorded.

However, despite the importance of the construction industry for national economy, construction activities sadly pose serious H&S risks to workers, users of construction facilities and the public. A report by the International Labour Organisation (ILO) [5] acknowledged that the construction industry contributes about 30 percent of fatalities in the world’s occupational settings. Other researchers have corroborated that the construction industry has an infamous reputation as being one of the most dangerous sectors in the world [6,7,8]. Other studies reveal the occurrence of relatively high proportion of accidents on construction sites in developing countries [9,10,11].

A study by Hamalainen et al. [12] asserted that the rates of occupational accidents in these developing countries are unacceptably high and predict that the rates will increase parallelly with the pace of industrialisation. In Ghana, the Ghana Statistical Service [4] reported that in 2015, occupational injuries occurred at a frequency of 43 per 1,000,000 hours worked, with an incidence rate of 63 injuries per 1000 workers and severity rate of 418 days lost per 1,000,000 hours. Moreover, Hamalainen et al. [9] found that the occupational accident fatality rate in low- and middle-income African countries such as Ghana is 21.1 fatalities per 100,000 workers, which was far worse than the rates in developed countries as compared in Table 1. It must be noted that the accident and fatality rates in reality are likely to be much worse in developing countries, such as Ghana, considering that many non-fatal accidents in workplaces go unreported [13].

A plethora of studies have considered factors affecting H&S performance of the construction industry in developing countries [17,18,19,20,21,22,23]. Many of these studies provide evidence of challenges in managing H&S in the construction industry. Some challenges identified are weak regulatory systems, poor management commitments, lack of H&S training, lack of H&S procedures, inefficient enforcement [22] and lack of national framework for managing H&S [21], among others. For instance, Durdyev et al. [18] studied key factors affecting construction safety performance in developing countries by taking evidence from Cambodia. Through literature review, their study identified 30 factors that affect H&S performance of the construction industry in developing countries. These factors included among others, lack of training, reckless operations, poor equipment, lack of skilled labour and lack of personal protective equipment. Moreover, Kheni et al. [23] looked at the H&S management in developing countries by considering the practices of construction small and medium enterprises (SMEs) in Ghana. Their study found that few construction SMEs implement health and safety measures to control risks on their project and that SMEs need to do more to ensure compliance with H&S laws. While all these studies provide in depth analysis and evidence of factors that affect effective implementation of construction H&S in developing countries, they do not consider the implications of the characteristics and foundation upon which the industry is built and operates. Therefore, this research posits that the structure of the construction industry in developing countries and its inherent characteristics expose the industry to H&S challenges. The aim of this research is, therefore, to identify, through an extensive literature review and empirical study, the peculiar structures and characteristics of the construction industry in developing countries and their implications for H&S. 

Following this introduction section (Section 1), the rest of this paper is organised into four more sections. The next section reviews literature relating to nine unique characteristics of the construction industry in Ghana and their implications on H&S. This is followed by the research methods and discussion of results in Section 3 and Section 4, respectively. Finally, Section 5 concludes the paper and makes recommendations.

## 2. Literature Review

Understanding the foundation and characteristics of the construction industry that impact on its H&S culture is key to improving the industry’s H&S performance. Zou [24] (p. 12) defined safety culture in construction *as an assembly of individual and group beliefs, norms, attitudes and technical practices that are concerned with minimizing risks and exposure of workers and the public to unsafe acts and conditions in the construction environment*. The H&S culture paradigm thus constitutes a holistic way of thinking about H&S management to reveal underlying factors affecting H&S performance of a complex system. Typically, culture relates to human experience and interaction, and this means that H&S culture within an industry is concerned with the systems, attitudes, behaviours and environmental factors that influence H&S performance. A study by Zou and Sunindijo [25] suggests that the characteristics and environment within which the construction industry operates influence the industry’s H&S performance. Peckitt et al. [26] emphasised this point by identifying the characteristics of the construction industries in the UK (a developed country) that negatively impacts on the industry’s H&S culture. Amongst others, the characteristics identified included complex regulatory system, focus on cost, intense competition, division between design and construction, mistrust and blame, short-termism, dominance of small contractors, prevalence of subcontracting, complex fragmented relationships and lack of consideration of H&S in design. In developing countries, Peckitt et al. [26] outlined characteristics such as absence of investment, inadequate construction regulations, lack of enforcement, limited resources, limited use of technology, materials supply problems, dominance of small companies, skills shortages and lack of training, job insecurity and others as the negative characteristics of the Caribbean construction industry safety culture. On the other hand, the features of positive H&S cultures within an industry or organization follow many aspects of total quality management, including risk elimination and control, empowerment, participation, humanistic values, teamwork, training and monitoring [26]. Considering that the characteristics and the environment of the construction industry have implications for the industry’s H&S culture and performance, the remaining section discusses the characteristics of the construction industry in Ghana and their impact on H&S. 

### 2.1. Colonial Influence

The structures, systems, practices and procedures of the construction industry in developing countries remain the same as those that were introduced by the former colonial countries [27]. From observations and reference to legal and regulatory documents such as the Building Regulations 1996, the Ghanaian construction industry derives its practice from the British construction industry [28,29]. However, Ofori [30] pointed out that, in developing countries, the existing construction project management systems that were inherited from the former colonial administrators bear no relation to the local culture, administrative systems and authority structures. In addition, Rwelamila et al. [31] argued that the lack of cultural appropriateness of procurement systems/methods is a factor that accounts for the poor performance of construction projects in Southern Africa. While major changes have been made in developed countries, those in developing countries mostly remain unchanged. For example, Ghana, which is a former British colony, mainly uses standard forms of contract that are comparable, or the same as, the 1973 edition of the Joint Contracts Tribunal form. Other public-sector forms of contract also remain the same as those drafted by the colonial architects and engineers [27].

Similarly, Kheni [22] investigated H&S management practices of small and medium construction enterprises in Ghana and discovered that occupational H&S regulations in Ghana are still heavily based on practices from its colonial past. He stressed that these regulations, procedures and processes have not been reviewed to reflect the cultural, social, political and economic changes that the country has gone through. Wells [32] raised questions about the relevance of continuing the use of H&S regulations and procedures that are rooted in the past. For this reason, Ofori [30] has advocated for H&S systems that reflect the cultural, economic, political and social changes that have taken place within the country over recent decades.

### 2.2. Procurement Systems

The traditional procurement system inherited from the British system is still the most popular form of procurement route for many projects in Ghana. The popularity of this procurement method in the construction industry in Ghana is mainly due to the predominance of the public sector and the demand for openness and public accountability. The clients’ familiarity with the system is another factor that contributes to its popularity. However, despite its popularity, there is a counterargument that shows the problems with using the traditional procurement method, including time-consuming, discourage innovation, decreased buildability and the fragmentation of the project teams [33,34]. According to Rwelamila and Smallwood [35], the traditional procurement method does not complement H&S due to the separation of the design and construction processes. 

Generally, the method of project procurement in the construction industry in Ghana has been in the form of competitive tendering. The system was further reinforced by the promulgation of the Public Procurement Act in 2003 (Act 663), which requires all public sector contracts (except in exceptional situations) to be awarded through the process of competitive tendering with a focus on tender price. Competitive tendering, together with the practice of awarding contracts based on the tender cost, results in a fierce competition among the large number of construction businesses in the country. Consequently, this may result in under-pricing by many construction firms in a bid to win contracts and subsequently not being able to perform upon award of the contract [36]. Besides, lowest price is not the best approach to achieve the overall lowest cost upon project completion [37]. 

A study by Smallwood [38] argued that competitive tendering marginalises H&S in construction. He remarked that, for instance, in South Africa, due to competitive market conditions, construction firms commonly find themselves in the iniquitous position that if they make the requisite allocations for H&S, they run the risk of losing a tender or negotiations to a less committed competitor. Similarly, Ngowi and Mselle [39] posited that this practice within the industry is a disincentive to the effective management of H&S. They remarked that in developing countries, contractors gain little competitive advantage from good H&S management because the industry culture compels contractors to drive their prices low. In this case, a way to cut costs is to cut corners when it comes to H&S.

### 2.3. Huge Number of Informal Sector Participation

The Ghanaian construction industry is divided into two sectors: the organised, “formal”, and the unorganised, “informal” sectors of the industry. The formal sector is based on the institutional arrangement and regulatory systems [22]. The sector is made up of firms that are legally registered and carry out organised construction projects with a combination of skilled workers, labourers and/or expatriates. The formal sector operates under set rules and regulations, including adherence to national laws on employment, procurement and H&S. Furthermore, the government is aware of all the activities of the sector and collects taxes from the firms.

Informal sector consists of units engaged in construction activities with the primary objective of providing employment and incomes to the individuals involved. These units usually operate on a small-scale basis, with low levels of organisation and with little or no division between labour and capital as factors of production. Labour relations, where they exist, are based mostly on casual employment, kinship or personal and social relations rather than contractual arrangements with formal guarantees [4]. This sector mostly consists of small builders and clients seeking to carry out construction of single dwelling houses for their families. 

According to Wells [40], an increasing number of private clients in developing countries choose to bypass general contractors and the more formal processes for awarding contracts, in favour of buying materials and managing the process themselves, while engaging directly with informal sector enterprises to supply labour. Contracts between the parties are mostly verbal, and the building process takes place in stages. Wells and Wall [41] referred to this method of organising the construction process, without the use of contractors or formal contracts, as the “informal construction system”. Clients using this informal procedure to construct their houses usually buy materials in small quantities, as and when they have the funds. The popularity of the informal construction process has also expanded the market for small producers and suppliers of construction materials. 

These informal sector enterprises are mostly not registered according to national or local governments regulations, and workers fall outside of the framework of labour regulation and do not enjoy any legal protection or entitlement to certain social benefits, such as sick leave, maternity leave and annual leave. Consequently, regulations or legislations, such as those relating to labour, conditions of employment and H&S, for the informal construction workers are generally flouted [40]. According to Ishfaq et al. [42], informal sector construction workers are predominantly illiterate, lack understanding of occupational health and safety (OHS) laws and therefore do not generally implement safety procedures on their construction sites. Moreover, the government has insignificant influence on the operations of the informal sector and receives little or no revenue through taxes; hence, it is very difficult to obtain reliable statistical data about the informal sector and monitor the H&S performance of those operating within it.

### 2.4. Large Number of Small Contractors

The construction industry in Ghana is characterised by a multiplicity of small firms [43] because the industry presents little barriers to entry, and this allows even individuals and business entities without the requisite resources, personnel or qualifications to register as construction firms [44]. Ayisi [45] opined that the more than 20,000 building construction firms registered with the Ministry of Water Resources, Works and Housing (MWRWH) are a relatively huge number, given the size of the Ghanaian economy. Moreover, figures from the MWRWH put the registered contractors at about 34,000 in 2010 [46]. A study by Egmond et al. [43] revealed that out of a total of 7095 construction firms registered in 2002, ninety percent are small contractors and undertake less complex construction jobs. The total amount of work executed by these small firms ranged between 10 percent and 20 percent of the total construction output [47]. Such firms cannot compete with foreign contractors who are much better equipped to capture a major share of the local construction market, especially when it concerns more complex projects [43,47]. Addo-Abedi [48] reported that most of the small and medium-sized firms are domestic contractors, and they are managed as family-run businesses.

In another vein, it is acknowledged that small contractors generally do not manage H&S risks as effectively as larger contractors [49] and, consequently, suffer higher risks of occupational accidents than larger firms [23,50]. Sunindijo [51] studied key barriers and strategies for improving safety among small construction organisations and revealed that large contractors demonstrate good safety performance because they have the resources and leverage to develop and implement robust safety management systems, but small firms are far behind. The issue of cost is seen as the key factor affecting H&S performance of the small contractors. For instance, buying personal protective equipment (PPE) and employing safety officers on projects invariably means additional costs [52], but with the size and capital of these small contractors, the resources and facilities to enable safe construction are not readily available [52,53]. Other authors including Pinto et al. [54] reinforced this point by stating that the financial fragility and instability of small contractors can impede the extent to which good H&S practices can be adopted. For these small firms, business survival is their top priority, and therefore, H&S issues are not of great concern [55].

### 2.5. Fragmented Industry 

Furthermore, the industry is known to be complicated and fragmented [28] involving many stakeholders such as clients, the design team and the construction team among others [56]. The construction environment is characterised by the separation between design and construction with professionals tend to operate independently [57]. This has resulted in the adversarial relationships that traditionally characterises the construction industry [58]. Fundamentally, fragmentation is inherent in the traditional procurement system (which is the system most often used in Ghana). With this system, the design and construction of projects are executed by various parties and are therefore separate. The design team, which is made up of consultants (architects, engineers, etc.), produces designs, provides details of materials, products and how the works should be performed. The contractor (with the help of suppliers and sub-contractors) would subsequently execute the tasks according to the design. The fragmentation in the construction industry is characterised by lack of a sense of identity, promoting a confrontational culture and lack of feedback loops or coordination between the design and construction [59,60,61]. There is a lack of interaction between consultants/designers and contractors during the design and construction phase, and this set-up often affects project outcomes during the construction phase such as increased project complexity, rework, cost and time overruns as well as safety concerns [62]. According to Donaghy [63], one causal factor in construction industry fatalities is the separation of, and poor communication between, design and construction functions. The traditional system typically defines the roles, responsibilities and liabilities of different players involved in a construction project [64]. This tends to isolate the consultants by seeing them as a stand-alone party. In this environment, the consultants assume that there is no direct benefit to them from making their designs safer. Consequently, many H&S issues that should have been dealt with during the design stage, only surface during the construction, operation and maintenance phases of the project [8]. 

According to the Commission of the European Communities [65], over 60 percent of all fatal construction accidents can be attributed to decisions made before commencing work on site. This suggests that decisions made early in a project’s life, especially during the design stages, may influence H&S of site operatives who will build the project according to the design and specifications provided by the consultants [8]. Considering H&S in the design stages of projects is an important approach for safety management, because it is an effective way of either reducing or eliminating hazards at their sources. However, the separation of design responsibilities from the responsibility to construct denies contractors the opportunity to provide input. This can seriously limit the identification of innovative solutions to H&S problems at the design stage of projects [8]. Hinze and Gambatese [66] posited that incorporating contractors’ experience and knowledge at the design stage can improve project “constructability” and eliminate H&S problems at source. Fragmentation affects constructability of project and this leads to increased H&S risks during construction, operation and maintenance of projects.

### 2.6. Lack of Single Regulatory Authority

Besides the highly fragmentary structure, the other most noticeable feature of Ghana’s construction sector, which perhaps best explains its problems, is the lack of co-ordination and absence of a clear agenda and a central agency to regulate and ensure the continuous development of the industry [29,30,67,68,69]. There is no single government agency in Ghana that oversees the construction sector. Responsibility for and jurisdiction over the built environment are shared between MWRWH and Ministry of Roads and Highways (MRH). Besides, the Ministry of Employment and Labour Relations (MELR) deals with the labour and employment aspects of the sector, while the Ministry of Education partly deals with vocational/technical training, research and development. 

According to Ofori-Kuragu et al. [46], the somewhat inconsistent and ad-hoc nature of construction policies reflects the way responsibility for the sector is divided across these ministries. The non-existence of a central body within the construction industry is a disincentive to the enforcement of H&S regulations within the industry. For instance, in Hong Kong, the Construction Industry Council (CIC) has been established as a statutory body and among their functions, to promote good practices in the construction industry in relation to occupational safety and health, procurement methods, project management and supervision, dispute resolution, environmental protection, etc. The CIC regularly monitors and reviews the safety performance of the construction industry in order to institute measures for improving health and safety within the industry. It has authority to formulate and enforce codes of conduct on matters such as health and safety. Contractors who fail to adhere to lawful directives issued by CIC shall be guilty of an offence. This kind of statutory arrangement is absent in the Ghanaian construction industry, and as a result, enforcement of H&S standards on construction sites is weak.

### 2.7. Reliance on Labour Intensive Methods

The construction industry in Ghana, like many other developing countries, relies on labour intensive methods. Typical infrastructure, for example, feeder roads, small-scale irrigation systems, buildings, small dams, among others, are constructed using labour-based methods [22]. Labour in Ghana is comparatively cheap, making the adoption of labour-based methods a more economic option than equipment-intensive or capital-intensive methods. Kheni [22] mentioned that the capital cost of acquiring equipment and/or machinery is a hefty one for contractors in Ghana considering the problems they face in accessing credit facilities for such items, and this compels many, especially the smaller contractors, to specialise in labour-based construction methods. Accordingly, Koehn et al. [70] asserted that construction in developing countries like Ghana involves more workers per activity on site, typically, 2–10 times as many workers per activity compared with developed countries. This means that more site operatives are exposed to H&S hazards. 

### 2.8. Reliance on Temporary Labour Force

Construction firms in Ghana like many others throughout the world have only a small core of regular workers while bulk of its field workers are employed on a temporary and casual basis [22,40,71]. Wells [40] attributed this phenomenon (of firms shedding their permanent labour force and replace them with temporary and casual workers) to increased competition within the industry, declining workloads and/or restrictive employment regulations.

These temporary workers often do the same job and carry out the same tasks as permanent employees, but they do not receive the same level of training or work conditions (e.g., bonuses, promotion, etc.). According to Morrison [72], temporary workers face higher risks on the job due to a lack of training and a perception that they could be seen as “disposable”. This is reinforced by Dawson et al. [73] when they asserted that temporary workers belonged to a category of workers who are “at risk” in the workplace. Ideally, these workers need to be trained to perform their jobs safely, to recognise, understand and avoid potential hazards to themselves and others [74]. However, many temporary and casual workers have less access to training particularly on H&S, as the contractors may not be willing to invest to train non-permanent workers. 

A study by Mitullah and Wachira [75] emphasised that these kinds of workers do not belong to any form of labour union, and this makes it difficult for them to compel their employers to adhere to good labour and safety standards. Again, the abundance of cheap labour in developing countries means that the construction firms can dismiss employees who perform unsatisfactorily and replace them with new employees easily. This causes them to accept work in unacceptably high-risk situations without complaining or demanding that their employers put in place H&S measures, and this leads to many accidents on site [70].

### 2.9. Lack of Skilled and Educated Workforce

The construction industry in developing countries like Ghana has inadequate supply of skilled, educated and experienced workforce. A survey within the Ghanaian construction industry revealed that among the workforce involved in the sector, 67.2% are unskilled, 24.8% are semi-skilled and 8% are highly skilled [76]. This means that, despite the availability of plentiful cheap labour at the disposal of the construction industry in Ghana, demand for construction skills is only partially met, especially for artisans and tradespeople [77]. This has been attributed to the limited capacity of existing technical and vocational training institutes in terms of small class sizes, workshops, insufficient teaching and administrative personnel and outdated curriculum [78]. Again, the informal apprenticeship system is not well developed to train highly skilled workforce because sometimes the master craftsmen who do the training may themselves have limited skills. The need for the development of a skilled workforce for construction industries of developing countries has been acknowledged [3,79].

In addition, many of the workers on construction sites in developing countries are barely literate, and these illiterate employees are often difficult to convince on matters relating to H&S [22]. What is often important to illiterate workers on the construction sites is the wage that they would earn for working on site, thereby making other issues relating to conditions of employment secondary to them [22]. Similarly, Koehn et al. [80] have emphasised that a key barrier to H&S management is the difficulty in training illiterate workers.

While the construction industry is hailed as an avenue for employing unskilled labour, this has partly contributed to its negative image of having poor H&S record. Cooper and Cotton [81] posited that the lack of skills and education of construction operatives is a major cause for construction site accidents. Similarly, the lack of training for these workers on H&S matters worsens the situation [69]. For that matter, Darko and Lowe [3] and Frimpong et al. [79] have advocated for the provision of training programmes for construction artisans in Ghana in order to provide them with the necessary skills required for efficient and safety performance at the construction sites.

## 3. Research Method

This research study adopted quantitative research methodology. Quantitative research is used to verify a theory rather than develop one, by applying the principles of deductive reasoning. Generally, quantitative research comprises the collection and analysis of data through statistical procedures. Mostly, this analysis is done with the aim to determine the truth or otherwise of hypotheses or theory [82]. Therefore, this method is appropriate to answer the questions about how the characteristics of the construction industry in developing countries influence H&S management.

The study included a population of professionals in the construction industry in Ghana who have direct and indirect involvement in H&S management on projects. These participants included contractors, consultants, clients of public projects, trade unions/ associations and suppliers. The participants were from different professional backgrounds in the construction industry including engineering, quantity surveying, architecture, project management, occupational H&S, building technology, etc.

A total of 110 questionnaires were administered to construction professionals working in various construction organizations in Ghana. The choice of participants and distribution of 110 questionnaires stemmed from assurances given by some respondents on one of the researchers’ ongoing studies. To be exact, in an ongoing study by the researchers into H&S performance improvement in Ghana, 110 out of the total respondents in that ongoing study indicated their willingness to participate in this research. This resulted in the convenient choice of the research participants and the distribution of 110 survey questionnaires. Although convenience sampling method may be prone to sampling error and lack of representation of population, these participants are spread across 5 typical regions of Ghana, with different professional backgrounds and varied levels of construction industry experience. In total, 46 complete/valid responses were received, and 3 responses were discarded due to incomplete responses. This represents a relatively high response rate at about 44.5%.

The survey questionnaires were designed to seek the views of professionals within the Ghanaian construction industry, on the extent to which some identified characteristics of the construction industry in Ghana affect H&S management within the industry. The questionnaires were made up of two sections. Section one collected details of research respondents including their professional background, number of years of experience in the construction industry and the nature of their organisations. Section one consisted of nine key characteristics of construction industries in developing countries that have been identified through the literature. This section measured the level of agreement of industry professionals on how the identified characteristics affect H&S management. Likert scale was used in the questionnaire with ranges between 1–5, where 1 = strongly disagree, 2 = disagree, 3 = neutral, 4 = agree and 5 = strongly agree. Content validity was achieved by ensuring that analysis was carried out based on issues identified in the literature, which were tailored to match the construction industry in Ghana.

## 4. Results and Discussion

### 4.1. Background of Survey Respondents

Table 2 shows the profile of respondents. On the professional background, it indicates that out of the 46 respondents, 23.9% have Engineering background, 15.2% have Quantity Surveying background, 13.0% Architecture, 15.2% are Project Management professionals, also 15.2% are Occupational H&S experts, 6.5% are Building Technologists and 10.9% have other professional backgrounds. With respect to the number of years’ experience working in the construction industry, 4.4% of the respondents have experience ranging from 1–5 years, 13.0% have 6–10 years, 39.1% have experience ranging between 11–15 years, 32.6% have 16–20 years and 10.9% have experience of over 20 years. Again, Table 2 shows the type of organisations within the construction industry where the respondents work. It shows that 28.3% of the respondents work as consultants, 37.0% are contractors, 23.9% work for government institutions, 6.5% are from trade unions/associations, 2.2% are suppliers of construction products and 2.2% work with other construction industry organisations not listed.

### 4.2. Ranking of Identified Characteristics

Table 3 presents the perceptions of the respondents on the extent to which characteristics of the construction industry in developing countries, particularly Ghana influence H&S management within the industry. In general, the respondents agree that the peculiar characteristics of the construction industry in Ghana have a negative influence on the management of H&S within the industry, as indicated by the total average of 3.75. The top three characteristics are lack of skilled and educated workforce, reliance on labour intensive methods and the lack of single regulatory authority overseeing the construction industry. The characteristics that have the least negative influence on the management of H&S are the fragmented practices in the industry and colonial influence.

Overall, respondents strongly agreed that the lack of skilled and educated workforce within the construction industry has the most negative influence on the management of H&S within the industry. This emphasises the point that many construction site operatives in developing countries are unskilled and/or illiterate and often difficult to manage on matters relating to H&S [22]. Respondents believe that the matter of the construction industry being an employment avenue for many unskilled and illiterate workers presents a huge challenge to H&S management. In a related matter, respondents are of the view that the reliance on labour intensive methods of construction has a huge influence on OHS management. Many contractors do not have the appropriate plants and equipment, as well as the technologies to safely carry out their tasks. Consequently, many contractors adopt labour-intensive methods that result in more workers per activity on site. For instance, a contractor working without a concrete mixer may employ around 10 labourers to mix say 15m^3^ of concrete per day, a task which could have been done by 3 workers if there was a concrete mixer. As a result, more workers (mostly unskilled and uneducated) are exposed to H&S hazards. 

Moreover, the lack of a single regulatory authority to oversee the construction industry in Ghana was highly rated by respondents as having a negative influence on H&S management within the industry. There is little enforcement of H&S regulations in the industry partly due to the lack of the single regulatory authority. Similarly, the laxity in enforcement of H&S regulations is partly responsible for the poor H&S performance of the industry. In essence, respondents agreed that enforcement is key to achieving better H&S in the industry, as it ensures compliance with H&S regulations and reduces the risk of work-related accidents and illness to acceptable levels [8]. On the other hand, respondents were virtually neutral on the influence of the fragmented nature of the industry. This reinforces the point that, though this characteristic of the construction industry may have significant influence on H&S management, it is not limited to developing countries alone. 

A comparison of the responses given by the various organisations is also shown in Table 3. The responses from consultants, contractors and government organisations are specifically highlighted. It is noted that the average responses of these three organisations are relatively close, although the rankings are not the same in all instances. This indicates that these different organisations do not differ significantly in this belief about how the various characteristics of the construction industry influence H&S management. However, although the fragmented nature of the industry was ranked last by all these organisations, the average response given by consultants indicates that their organisation essentially disagrees that it has a negative influence on H&S management.

### 4.3. ANOVA Results and Discussion 

An analysis of variances (ANOVA) has been done in order to determine whether the nature of the respondent’s organisation has an influence on their responses. Table 4 presents the results of the ANOVA following the responses obtained from consultants, contractors and government institutions. Prior to performing the ANOVA, skewness and kurtosis index were used to identify the normality of the data. The assumption of normality was evaluated and determined to be fulfilled as the distributions were associated with skewness value of −1.07 to 0.43 and kurtosis value of −0.69 to −0.13 (see Table 3). According to Byrne [83], normality of data could be assumed if the skewness and the kurtosis values ranges between −2 to +2 and −7 to +7 respectively. Furthermore, the assumption of homogeneity of variances was tested based on Lavene’s F test, and the results showed that 2 variables (CHA2 and CHA7) violated the homogeneity of variance assumption needed for a regular ANOVA (see Table 5). Therefore, the Welch ANOVA test was performed instead of the regular one-way ANOVA test.

From Table 4, the results of the Welch ANOVA test between the three organisations revealed that there were statistically significant differences in the responses for CHA5- fragmented industry (F(2,21.252) = 3.553, *p* = 0.047). On the other hand, there were no statistically significant differences in the responses of the organisations in respect of the remaining challenges. Consequently, in order to determine the source and evaluate the nature of the differences between the responses of the organisations in the statistically significant ANOVA, Games–Howell’s post-hoc multiple comparison test (for equal variances not assumed) was performed and the results presented in Table 6. 

The post-hoc comparison on CHA5 indicated that the differences between the responses from consultants and contractors were statistically significant (MD = −0.756, *p* = 0.037); however, the responses between consultants and government institutions (MD = −0.462, *p* = 0.374), and contractors and government institutions (MD = 0.294, *p* = 0.542) were both not statistically different. The results indicate that consultants and contractors differed significantly in their beliefs about how the fragmented nature of the construction industry in Ghana has resulted in H&S challenges, with contractors showing higher levels of agreement to the challenges posed by the fragmentation (CHA5), much more than consultants did. 

It is acknowledged that fragmentation results in the separation of design and construction phases, with consultants being the central figures on projects. Several studies [84,85] have questioned the attitude of consultants to other conventional procurement methods that eliminate the separation of design and construction stages. Consultants may perceive that delivery methods other than the traditional design-bid-build as threats to their profession as their traditional leading roles and decision-making powers on projects are taken away and mostly given to contractors. This difference may also be explained by the party that is most affected by fragmentation. Contractors are responsible in constructing facilities based on design drawings. When a design has constructability issues and is difficult to be constructed, this can affect H&S in the project. This issue may be obvious to contractors, but designers who are not usually involved in the construction process may not appreciate the significance of the matter. The reasons above may have influenced the different perceptions shared by consultants and contractors on the H&S impact of the industry fragmentation.

### 4.4. Correlation Analysis and Discussion

The relationship between the years of industry experience and the various identified characteristics of the industry was analysed using the Pearson’s product–moment correlation coefficient. The number of years, which is a proxy of the amount of experience in the industry, influences the way construction practitioners make decisions. It is posited that this experience serves as a learning platform for construction practitioners to appreciate the norms and characteristics of the industry, thus allowing them to operate within constraints of these norms and characteristics. Previous studies have shown that education, age and work experience are key determinants of H&S perceptions, hazard identification and risk management [86,87,88]. Therefore, correlating the number of years’ experience with the identified characteristics provides an idea about how the beliefs and perceptions of the industry professionals have been shaped by their level of professional experience within the industry. 

In Table 7, the matrix showed three significant correlation between the variables. The results showed that there is a strong negative correlation between years of industry experience and the fragmented nature of industry (r = −0.512, *p* ≤ 0.01). This essentially means that the more experienced the respondent is, the lesser the level of agreement with the H&S challenges posed by the fragmentation in the construction industry. The fragmentation is typically associated with the traditional procurement system, which is the dominant and popular procurement method in the construction industry in Ghana. Therefore, the more one is experienced in the industry, the more the person is familiar with this practice and may not recognize it as a significant challenge to H&S performance within the industry. 

Moreover, there is a positive correlation between colonial influence and lack of single regulatory authority (r = 0.427, *p* ≤ 0.01), which essentially means that the respondents who agreed that colonial influence within the industry influences H&S performance, also agreed to the challenges posed by the absence of a single regulatory authority and vice versa. It is acknowledged that the construction industry in Ghana is heavily based on the foundations laid in the colonial era [27], which did not make provision for a single regulatory body to oversee the industry. Accordingly, the more the industry continues to depend on the systems, procedures and practices which are rooted in the past, the more it is going to lack an established regulatory body to champion the industry’s growth and improvement including H&S. 

Finally, the matrix shows a negative relationship between colonial influence and reliance on temporary workforce (r = −0.364, *p* ≤ 0.05). This shows that a significant number of respondents who expressed higher levels of agreement to the effect of colonial influence on H&S performance in the construction industry did not agree to the H&S impact of the reliance on temporary workforce and vice versa. Admittedly, the presence of a huge number of contractors has resulted in heightened competition for jobs in the construction industry and decreased workload for many contractors. This together with other factors such as restrictive labour and employment regulations have caused many contractors to rely on temporary and casual workforce in recent times [40]. This phenomenon was not the practice in the past, and this may have accounted for this significant negative relationship between the two characteristics.

## 5. Conclusions

This paper has considered the H&S implications of the peculiar characteristics of the construction industry in Ghana. This stems from the premise that the influence of the peculiar structure and characteristics of the construction industry in developing countries on H&S cannot be underestimated. Within the context of Ghana, the identified characteristics included lack of skilled and educated workforce, reliance on labour intensive methods, lack of single regulatory authority, huge informal sector participation and proliferation of small contractors. Also identified are the dominance of traditional procurement system, reliance on temporary workforce, colonial influence and fragmentation within the industry. Awareness of these characteristics (and their impact on H&S) is necessary for a complete understanding of H&S management within the construction industry in developing countries. Remedying the challenges that these characteristics present is key to managing construction H&S.

The findings from the research indicate that significant actions are required to change some of the characteristics of the construction industry in Ghana in order to improve its H&S performance. The study identifies the lack of skilled and educated workforce, reliance on labour intensive methods and lack of single regulatory authority as the top three characteristics that have the highest negative influence on H&S performance of the industry. Unskilled, uneducated and untrained workers on the construction site can easily cause accidents, because this group of workers are often difficult to persuade on matters relating to H&S. Hence, there is the need to train these unskilled construction workers in Ghana in order to provide them with the necessary skills required for efficient and safe performance at construction sites. Consequently, it is recommended that anyone who wishes to work in the construction industry in Ghana must have some level of certification issued by authorised agencies, which will attest to their level of skills and competencies to execute tasks effectively and safely. This can be done after the candidate has gone through some level of training and attained the required level of competence and then registered. For instance, in Hong Kong, no one is permitted to work on a construction site unless he/she is duly registered by the Construction Industry Council (CIC). Adopting this kind of arrangement will ensure that site workers have some level of training and skills. 

The reliance on labour intensive methods in the construction industry in Ghana directly exposes many site workers to the risks associated with their tasks. Many construction organisations mostly rely on labour-intensive methods because they do not have the requisite plants and equipment, as well as the technology to perform those tasks. While labour intensive construction methods create opportunities for a considerably larger number of unskilled and low-skilled jobs than if equipment-based methods were used, they also expose more workers to hazards, and this has resulted in the poor safety performance of the industry. This study recommends that credit worthy contractors be supported by government to procure plants and equipment to use on their projects. On the other hand, where a project’s contract condition specifies the use of labour-intensive method, it is recommended that the contractor and safety authorities should take effective actions to ensure proper safety implementation through education and enforcement.

The lack of a single regulatory authority to regulate the operations of the construction industry has been identified as a major factor affecting the safety performance of the industry. A single authority is important to ensure that activities of the industry are properly streamlined and regulated. It is advocated that this body must be instituted as a statutory authority backed by an Act of Parliament and be well-resourced to carry out its mandate. Following the examples of the Construction Industry Council (CIC) in Hong Kong and the Construction Industry Development Board (CIDB) in Malaysia and South Africa, Ghana can establish its statutory authority with a mandate to regulate the activities of the construction industry and to ensure its continuous improvement in all areas including H&S. To this end, this research joins the call for a single regulatory authority to oversee the construction industry in Ghana.

This study contributes not only to academic research on H&S performance improvement in developing countries but also points out the fundamental challenges confronting the construction industry in Ghana to adequately manage H&S. While many previous studies have provided in depth analysis and evidence of factors impacting on construction H&S management in developing countries, none has fully considered the H&S implications of the characteristics and foundation upon which the industry is built and operate. In filling this research gap, this study through literature review and empirical investigation has identified the peculiar structures and characteristics of the construction industry in developing countries and their implications for H&S. The recommendations provide practical insights to government and professionals within the industry to tackle the basic problems that have resulted in the industry’s poor H&S performance. As a case study, data was collected from Ghana; therefore, the results are mainly relevant to Ghana. Nevertheless, the findings of this research could be used as a framework for country-specific studies in other developing countries. Literature has shown that the construction industry in developing countries shares similar characteristics, some of which have been discussed in this research along with their implications for H&S management. Typically, there is little difference in technology, procurement systems, construction methodology, skills training and availability, as well as poor H&S performance among developing countries. Therefore, the findings of this research may be applicable to many other developing countries. Moreover, considering that the characteristics of the construction industry have influence on H&S management within the industry, future research can identify more of these characteristics.

## Figures and Tables

**Table 1:** Occupational accident fatality rate for different countries.

Country	AFRO * Region Such as Ghana	Australia	UK	France	Germany	USA
Accident fatality rate per 100,000 workers	21.1	1.5	0.55	3.14	0.81	3.6

* Low- and middle-income countries of the African Region (AFRO). Sources: For AFRO regions such as Ghana [9], for Australia [14], for UK, France and Germany [15] and for the USA [16].

**Table 2:** Profile of respondents.

			Quantity	Percentage (%)
1	Professional background	Engineering	11	23.9
		Quantity Surveying	7	15.2
		Architecture	6	13.0
		Project Management	7	15.2
		Occupational health and safety	7	15.2
		Building Technology	3	6.5
		Other	5	10.9
2	Years of work experience	1–5 years	2	4.4
		6–10 years	6	13.0
		11–15 years	18	39.1
		16–20 years	15	32.6
		Over 20 years	5	10.9
3	Type of organisation	Consultant	13	28.3
		Contractor	17	37.0
		Government institution	11	23.9
		Supplier	1	2.2
		Trade union/association	3	6.5
		Others	1	2.2

Total number of respondents = 46.

**Table 3:** The extent to which characteristics of the construction industry in Ghana influence OHS management within the industry.

S/n	Characteristics	Consultants		Contractors		Government Inst.		Overall					
		Mean	Rank	Mean	Rank	Mean	Rank	Mean	Standard Deviation	Variance	Rank	Skewness	Kurtosis
1	Colonial Influence	3.23	8	3.41	8	3.45	5	3.37	0.73	0.54	8	−0.38	−0.53
2	Procurement system	3.69	6	3.82	4	3.45	5	3.65	0.76	0.57	6	0.08	−0.40
3	Huge number of informal sector participation	3.85	4	3.53	6	3.64	4	3.70	0.66	0.43	4	0.43	−0.69
4	Large number of small contractors	3.77	5	3.71	5	3.45	5	3.70	0.58	0.34	4	0.19	−0.54
5	Fragmented industry	2.54	9	3.29	9	3.00	9	2.98	0.77	0.59	9	−0.26	−0.49
6	Lack of single regulatory authority	3.92	3	3.94	3	4.09	2	4.00	0.63	0.39	3	0.00	−0.35
7	Reliance on labour intensive methods	4.31	2	4.41	2	4.09	2	4.24	0.52	0.27	2	0.24	−0.13
8	Lack of skilled and educated workforce	4.62	1	4.76	1	4.36	1	4.59	0.57	0.33	1	−1.07	0.22
9	Reliance on temporary labour force	3.69	6	3.47	7	3.36	8	3.50	0.77	0.6	7	0.29	−0.30

Number of respondents: Consultants = 13, Contractors = 17, Government institutions = 11, Overall = 46 (including Trade unions = 3, Suppliers = 1 and Others = 1); Note: 1 = strongly disagree, 2 = disagree, 3 = neutral, 4 = agree and 5 = strongly agree.

**Table 4:** Results of Welch ANOVA (robust tests of equality of means).

Welch	Statistic *	df1	df2	Significance
CHA1	0.336	2	24.118	0.718
CHA2	1.054	2	24.691	0.364
CHA3	0.949	2	23.655	0.401
CHA4	1.072	2	23.907	0.358
**CHA5**	**3.553**	**2**	**21.252**	**0.047**
CHA6	0.227	2	23.250	0.799
CHA7	2.368	2	24.763	0.115
CHA8	1.889	2	23.970	0.173
CHA9	0.674	2	23.583	0.519

* Asymptotically F distributed. Bold: Significantly different at 0.05 level.

**Table 5:** Test for homogeneity of variances (based on means).

	CHA1	CHA2	CHA3	CHA4	CHA5	CHA6	CHA7	CHA8	CHA9
Levene Statistic	0.825	5.351	1.908	0.036	1.311	1.371	11.933	0.438	0.921
df1	2	2	2	2	2	2	2	2	2
df2	38	38	38	38	38	38	38	38	38
Sig.	0.446	0.009	0.162	0.965	0.281	0.266	0.000	0.648	0.407

**Table 6:** Games–Howell HSD multiple comparisons.

Dependent Variable	(I) Type of Organisation	(J) Type of Organisation	Mean Difference (I–J)	Std. Error	Significance	95% Confidence Interval	
						Lower Bound	Upper Bound
**CHA5**	**Consultants**	**Contractors**	−0.756 *	**0.282**	**0.037**	−1.469	−0.042
		Gov’t Inst.	−0.462	0.337	0.374	−1.309	0.386
	Contractors	Consultants	0.756 *	0.282	**0.037**	0.042	1.469
		Gov’t Inst.	0.294	0.274	0.542	−0.407	0.995
	Gov’t Inst.	Consultants	0.462	0.337	0.374	−0.386	1.309
		Contractors	−0.294	0.274	0.542	−0.995	0.407

*: Significantly different at 0.05 level.

**Table 7:** Correlation results.

Pearson’s Correlation		Experience	CHA1	CHA2	CHA3	CHA4	CHA5	CHA6	CHA7	CHA8	CHA9
Experience	Correlation	1									
	Significance										
CHA1	Correlation	0.105	1								
	Significance	0.489									
CHA2	Correlation	0.036	0.192	1							
	Significance	0.814	0.201								
CHA3	Correlation	0.222	−0.037	0.093	1						
	Significance	0.137	0.805	0.538							
CHA 4	Correlation	−0.017	0.110	0.055	−0.015	1					
	Significance	0.913	0.466	0.715	0.922						
CHA5	Correlation	−0.512 ^a^	0.247	0.024	−0.187	0.228	1				
	Significance	0.000	0.098	0.872	0.215	0.128					
CHA6	Correlation	0.213	0.427 ^a^	0.092	0.106	0.119	0.000	1			
	Significance	0.155	0.003	0.545	0.483	0.432	1.000				
CHA7	Correlation	−0.282	0.282	0.156	−0.234	0.168	0.232	−0.134	1		
	Significance	0.058	0.057	0.300	0.118	0.264	0.121	0.375			
CHA8	Correlation	0.085	0.053	−0.08	0.013	0.079	−0.020	0.182	0.113	1	
	Significance	0.575	0.727	0.595	0.934	0.603	0.893	0.227	0.456		
CHA9	Correlation	−0.072	−0.364 ^b^	0.037	−0.215	0.048	−0.202	−0.180	0.136	0.171	1
	Significance	0.635	0.013	0.807	0.152	0.751	0.178	0.232	0.369	0.255	

^a^ Correlation is significant at the 0.01 level (2-tailed); ^b^ Correlation is significant at the 0.05 level (2-tailed). CHA—Characteristics, for instance CHA1 = Colonial influence.
